# Effects of Adenine Methylation on the Structure and Thermodynamic Stability of a DNA Minidumbbell

**DOI:** 10.3390/ijms22073633

**Published:** 2021-03-31

**Authors:** Liqi Wan, Sik Lok Lam, Hung Kay Lee, Pei Guo

**Affiliations:** 1 MOE International Joint Research Laboratory on Synthetic Biology and Medicines, School of Biology and Biological Engineering, South China University of Technology, Guangzhou 510006, China; wanliqi73@outlook.com; 2 Department of Chemistry, The Chinese University of Hong Kong, Hong Kong SAR 999077, China; hklee@cuhk.edu.hk

**Keywords:** DNA minidumbbell, DNA methylation, *N^1^*-methyladenine, *N^6^*-methyladenine, nuclear magnetic resonance spectroscopy

## Abstract

DNA methylation is a prevalent regulatory modification in prokaryotes and eukaryotes. *N^1^*-methyladenine (m^1^A) and *N^6^*-methyladenine (m^6^A) have been found to be capable of altering DNA structures via disturbing Watson–Crick base pairing. However, little has been known about their influences on non-B DNA structures, which are associated with genetic instabilities. In this work, we investigated the effects of m^1^A and m^6^A on both the structure and thermodynamic stability of a newly reported DNA minidumbbell formed by two TTTA tetranucleotide repeats. As revealed by the results of nuclear magnetic resonance spectroscopic studies, both m^1^A and m^6^A favored the formation of a T·m^1^A and T·m^6^A Hoogsteen base pair, respectively. More intriguingly, the m^1^A and m^6^A modifications brought about stabilization and destabilization effects on the DNA minidumbbell, respectively. This work provides new biophysical insights into the effects of adenine methylation on the structure and thermodynamic stability of DNA.

## 1. Introduction

**Figure 1:**
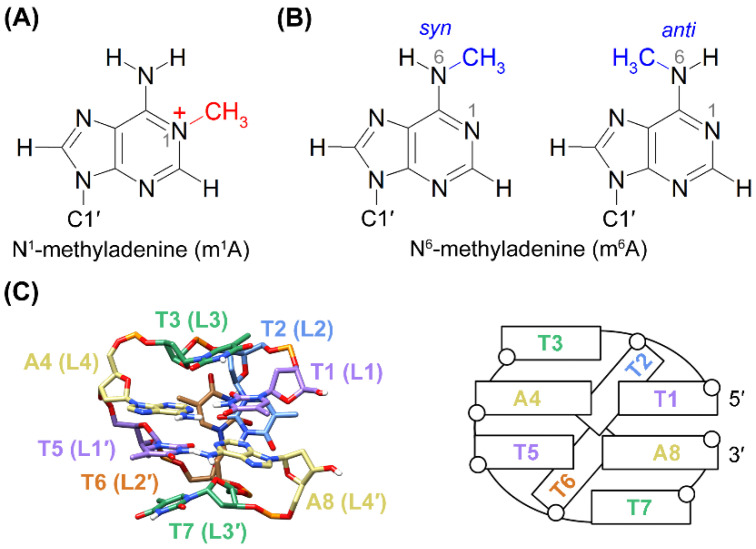
Chemical structures of (**A**) m^1^A, and (**B**) m^6^A with a *syn* and *anti N^6^*-methyl conformation, respectively. (**C**) An averaged nuclear magnetic resonance (NMR) solution structure (left) and a schematic secondary structure (right) of the TTTA minidumbbell (MDB) (PDB ID: 5GWQ) [23].

DNA methylation, where a methyl group is covalently incorporated to a nucleobase, plays an important role in gene regulations in prokaryotes and eukaryotes [1,2]. Methylation of adenine (A) can occur at the N1 or N6 site, resulting in the formation of *N^1^*-methyladenine (m^1^A) or *N^6^*-methyladenine (m^6^A), respectively (Figure 1A,B). The m^1^A is generated by endogenous and environmental alkylating agents in a single-strand DNA, and it blocks DNA replication if not corrected by DNA dioxygenases [3,4,5]. On the other hand, m^6^A occurs prevalently in prokaryotes as a part of the restriction–modification system [6,7]. Recent studies have also shown the existence of m^6^A in eukaryotes, though its presence in mammals remains disputable [2,8,9,10,11]. It is worth noting that m^1^A can be converted to m^6^A via the Dimroth rearrangement [4,12].

The effects of m^1^A and m^6^A on the structure and thermodynamic stability of DNA duplexes [13,14,15,16,17] and G-quadruplexes [18,19] have been investigated. Disruption of Watson–Crick base pairs by m^1^A has been well documented [14,16,17]. It is generally believed that steric hindrance of the *N^1^*-methyl group favors the formation of a T·m^1^A Hoogsteen base pair, in which m^1^A adopted a *syn* base orientation [17,20]. As a result, the thermodynamic stability of the DNA duplex was lowered due to the pairing of m^1^A with thymine [15]. For m^6^A, it can adopt two conformations, namely *syn* and *anti*, in solution depending on the position of the *N^6^*-methyl group relative to the N1 site (Figure 1B). The *syn* conformer of m^6^A was thermodynamically more favorable than the *anti* conformer such that the unpaired m^6^A showed a ≈20:1 preference for the *syn* conformation [21], which disrupted the Watson–Crick base pairing [18,21] and thus resulting in destabilization of the DNA duplex upon pairing of m^6^A with thymine [13,21]. On the other hand, unpaired m^6^A was found to stabilize the duplex [22] and G-quadruplex [19] structures through enhancement of stacking interactions. Yet, the effects of m^1^A and m^6^A on other types of DNA, especially those non-B DNA structures associated with genetic instabilities, remain unclear and worth further investigations.

Minidumbbell (MDB) is a recently discovered non-B DNA structure, which has been proposed to be one of the structural intermediates that causes genetic instabilities of short tandem repeats [23,24]. The first MDB structure was found to comprise two TTTA tetranucleotide repeats, which provides a possible pathway for one or two-repeat expansion mutations in *Staphylococcus aureus* variants [25]. The TTTA MDB is composed of two type II tetraloops, in which the first (L1/L1′) and fourth (L4/L4′) loop residues form loop-closing base pairs, the second loop residues (L2/L2′) are located in the minor groove, and the third loop residues (L3/L3′) stack on their nearby loop-closing base pairs (Figure 1C) [23]. Since then, similar MDB structures consisting of two CCTG tetranucleotide repeats [26] and ATTCT pentanucleotide repeats [24], respectively, have also been reported. The latter MDBs were suggested to associate with genetic instabilities in two human neurological diseases, i.e., myotonic dystrophy type 2 and spinocerebellar ataxia type 10, respectively.

The two T-A Watson–Crick base pairs in the TTTA MDB [23] and ATTCT MDB [24] serve as a core scaffold that stabilizes the corresponding MDB structure. Although the occurrence of adenine methylation on TTTA or ATTCT repeats in genomic DNA has not been reported and remains elusive, MDB can serve as a new non-B DNA model for studying the effects of adenine methylation on both the structure and thermodynamic stability of DNA. In this study, we used the TTTA MDB as a model to investigate the effect of m^1^A and m^6^A on the structure and thermodynamic stability of MDB. Two sequences were designed by substituting A4 in the TTTA MDB with m^1^A and m^6^A, i.e., 5′-TTTm^1^ATTTA-3′ and 5′-TTTm^6^ATTTA-3′ sequences, respectively. The NMR solution structures of 5′-TTTm^1^ATTTA-3′ were successfully determined, revealing that this sequence folded into an MDB containing a T1·m^1^A4 Hoogsteen base pair. Intriguingly, the thermodynamic stability of the 5′-TTTm^1^ATTTA-3′ MDB was slightly higher than that of the unmodified TTTA MDB. On the other hand, the 5′-TTTm^6^ATTTA-3′ sequence underwent a conformational exchange between two MDB conformers owing to the *syn* and *anti N^6^*-methyl conformations of m^6^A, and both conformers displayed lower stability compared with the unmodified TTTA MDB. Our results provide new insights into the effects of m^1^A and m^6^A on the structure and thermodynamic stability of non-B DNA.

## 2. Results

The solution structural behaviors of the two newly designed sequences, i.e., 5′-TTTm^1^ATTTA-3′ and 5′-TTTm^6^ATTTA-3′, were initially investigated using NMR spectroscopy. Sequential resonance assignments were made using standard methods from the H6/H8-H1′/H2′/H2′′ fingerprint regions of nuclear Overhauser effect spectroscopy (NOESY) spectra [27]. Sequential resonance assignments of 5′-TTTm^1^ATTTA-3′ and 5′-TTTm^6^ATTTA-3′ are shown in Figures S1 and S2, respectively. The H3′ signals of 5′-TTTm^1^ATTTA-3′ were assigned based on H1′/H2′/H2′′-H3′ correlations in the total correlation spectroscopy (TOCSY) spectrum, followed by ^31^P resonance assignments based on H3′-^31^P correlations in the heteronuclear single-quantum correlation (HSQC) spectrum (Figure S3) [27]. Adenine H2 signals of 5′-TTTm^1^ATTTA-3′ were assigned using long-range H2–C4 and H8–C4 correlations in the heteronuclear multiple bond correlation (HMBC) spectrum [28] (Figure S4). The ^1^H and ^31^P chemical shift information of 5′-TTTm^1^ATTTA-3′ is summarized in Table S1.

### 2.1. The 5′-TTTm^1^ATTTA-3′ MDB Containing a T·m^1^A Hoogsteen Base Pair

**Figure 2:**
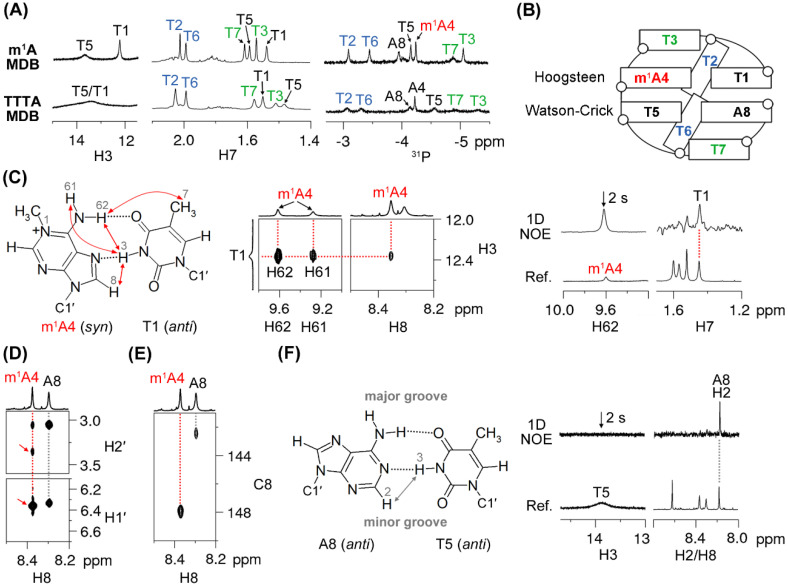
(**A**) A comparison of the H3, H7, and ^31^P signals between the m^1^A MDB and TTTA MDB [25]. (**B**) Schematic of the m^1^A MDB. (**C**) The T1·m^1^A4 Hoogsteen base pair is supported by NOEs of T1 H3-m^1^A4 H61/H62/H8 and 1D NOE of T1 H7-m^1^A4 H62. The *syn* base orientation of m^1^A4 is supported by (**D**) much stronger m^1^A4 H8-H1′ NOE than m^1^A4 H8-H2′ NOE (indicated by red arrows) and (**E**) downfield shifted m^1^A4 C8 signal in the ^1^H-^13^C HSQC spectrum. (**F**) The T5-A8 Watson–Crick base pair is supported by the 1D NOE of T5 H3-A8 H2. Spectra in (**A**,**D**,**E**) were acquired at 5 °C, and those in (**C**,**F**) were acquired at 0 °C. A mixing time of 50 and 450 ms, respectively, was used for the 2D NOESY spectra shown in (**C**,**D**), respectively.

We have recently reported the unique structural features of the TTTA MDB, including the folding of its T2/T6 into the minor groove as well as stacking of T3/T7 on the nearby loop-closing base pairs (Figure 1C), which resulted in unusually downfield shifted H6/H7/^31^P NMR signals of T2 and T6 and unusually upfield shifted H7/^31^P NMR signals of T3 and T7 [25]. The 5′-TTTm^1^ATTTA-3′ sequence shows similar downfield shifted T2 and T6 H6/H7/^31^P signals (7.90/2.03/–3.11 ppm for T2 and 7.83/2.00/–3.48 ppm for T6) and upfield shifted T3 and T7 H7/^31^P signals (1.53/-5.08 ppm for T3, and 1.61/-4.91 ppm for T7), which are comparable with those of the unmodified TTTA MDB (Figure 2A and Figure S1). Therefore, we believed that the 5′-TTTm^1^ATTTA-3′ sequence also folded into an MDB structure (named as “m^1^A MDB” in the following discussion) containing two type II tetraloops (Figure 2B). We noted that the T1 H3 signal (12.37 ppm) in the m^1^A MDB was much more upfield shifted than the T1 H3 signal (13.48 ppm) in the unmodified TTTA MDB (Figure 2A). A deeper analysis revealed that T1·m^1^A4 formed a Hoogsteen base pair in the m^1^A MDB, as evidenced by the NOEs of T1 H3-m^1^A4 H61/H62/H8 (Figure 2C). In addition, the observed 1D NOE of T1 H7-m^1^A4 H62 (Figure 2C) distinguished this Hoogsteen base pair from the reverse Hoogsteen base pair in which the methyl group of thymine is situated far from adenine H61/H62 [14,17]. In addition, the chemical shift of T1 H3 also agreed with those in Hoogsteen base pairs (11.5–13.1 ppm) [14,17,29]. In this T1·m^1^A4 Hoogsteen base pair, m^1^A4 adopted a *syn* base orientation, which is supported by (i) a stronger m^1^A4 H8-m^1^A4 H1′ NOE than the m^1^A4 H8-m^1^A4 H2′ NOE (Figure 2D) [30], (ii) an unusually downfield shifted m^1^A4 H2′ signal (3.35 ppm) (Figure 2D) [27], and (iii) a downfield shifted m^1^A4 C8 signal (147.88 ppm) (Figure 2E) [17,20,31]. In the m^1^A MDB, T5-A8 formed a Watson–Crick base pair, as evidenced by (i) the 1D NOE of T5 H3-A8 H2 (Figure 2F) and (ii) the appearance of T5 H3 at 13.87 ppm (Figure 2B). The slightly weaker A8 H8-A8 H1′ NOE than A8 H8-A8 H2′ NOE (Figure 2D) and the relatively upfield shifted A8 C8 signal (142.42 ppm) (Figure 2E) are also suggestive of an *anti* base orientation of A8.

We further determined the thermodynamic stability of the m^1^A MDB by performing ultraviolet (UV) melting experiments. It is interesting to note that the m^1^A MDB exhibited a melting temperature (*T_m_*) of ≈23 °C (which is higher than that of the original TTTA MDB by 4 °C) and a Δ*G°_25 °C_* value of 0.12 ± 0.04 kcal·mol^−1^ (as compared with that of 0.43 ± 0.08 kcal·mol^-1^ for the TTTA MDB) at 25 °C (Table 1). The lower Δ*G°_25 °C_* value for the m^1^A MDB is attributed predominantly to a more favorable change in entropy. Remarkably, the effect of the m^1^A residue on the thermodynamic stability of MDB was different from that on DNA duplex where a substitution of adenine with m^1^A led to destabilization [15]. It has also been reported that the accommodation of a T·m^1^A Hoogsteen base pair in a DNA duplex led to partial melting of neighboring Watson–Crick base pairs, as suggested by broadening of the corresponding NMR signals [17]. However, in the case of MDB, the resonance signal of T5 H3 in the neighboring T5-A8 Watson–Crick base pair appeared to be sharper in the m^1^A MDB than that observed for the TTTA MDB (Figure 2A). In fact, almost all of the proton signals of the m^1^A MDB were found to be sharper than those of the TTTA MDB. This phenomenon may be correlated with a higher thermodynamic stability and reduced local dynamics in the m^1^A MDB.

To gain deeper insights into the structural features of the m^1^A MDB, we calculated its high-resolution structures based on NMR restraints, including 333 NOE-derived distance restraints, four hydrogen bond restraints, 21 torsion angle restraints, and 40 chirality restraints (Table S2). Among 100 structures calculated by independent restrained molecular dynamics (rMD)–restrained energy minimization (rEM) experiments, five structures with the lowest restraint violation energies were selected as a final representative ensemble. The final structures agreed well with the NMR restraints with no distance and angle violation greater than 0.2 Å and 5°, respectively (Table 2). Covalent geometries have been reasonably maintained as revealed by the small deviations from ideal bonds distances and angles. An average pairwise RMSD of ≈0.6 Å reflected a good structural convergence among these five structures.

**Figure 3:**
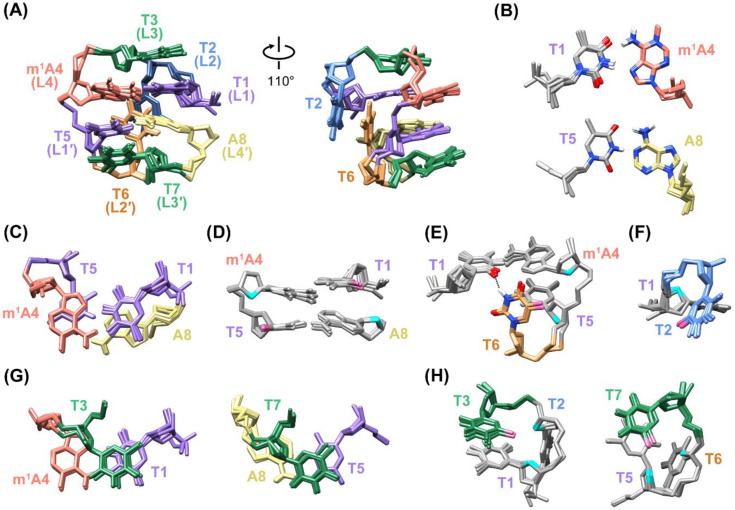
(**A**) The five superimposed solution structures of the m^1^A MDB (PDB ID: 7E4E). (**B**) T1·m^1^A4 Hoogsteen and T5-A8 Watson–Crick base pairs. (**C**) Base–base stackings between T1·m^1^A4 and T5-A8 base pairs. (**D**) Hydrophobic contacts between the methyl group (pink) of T1 and 2′-methylene group (cyan) of A8, and between the methyl group of T5 and 2′-methylene group of m^1^A4. (**E**) The T6 H3-T1 O2 hydrogen bond, and hydrophobic contacts between the methyl group of T6 and 2′-methylene groups of m^1^A4 and T5. (**F**) Hydrophobic contact between the methyl group of T2 and 2′-methylene group of T1. (**G**) Base–base stackings between T3 and T1·m^1^A4, and between T7 and T5-A8. (**H**) Hydrophobic contacts between methyl groups of T3/T7 and 2′-methylene groups of their two preceding residues.

Figure 3A shows the superimposed five representative structures of the m^1^A MDB (PDB ID: 7E4E). In this MDB, a T1·m^1^A4 Hoogsteen base pair and T5-A8 Watson–Crick base pair constituted the core scaffold with extensive base–base stackings (Figure 3B,C). The stackings between these two base pairs are also supported by multiple base–base NOEs of T1 H6-A8 H8, T5 H6-m^1^A4 H8, and m^1^A4 H2-A8 H8 (Figure S5A,B). Hydrophobic contacts were also observed between the methyl group of T1 and 2′-methylene group of A8, and between the methyl group of T5 and 2′-methylene group of m^1^A4 (Figure 3D), with the respective carbon···carbon distances falling within the range of 3.8–6.5 Å, which is favorable for the folding of secondary structures [33].

The location of T2 and T6 in the minor groove (Figure 3A) agreed with the occurrence of NOEs between their respective H7/H2′/H2′′ and A8 H2 (Figure S5C), as adenine H2 was pointing to the minor groove side in a Watson–Crick base pair (Figure 2F). In contrast, formation of the T1·m^1^A4 Hoogsteen base pair positioned m^1^A4 H8 in the minor groove. Therefore, the observation of NOEs of m^1^A4 H8-T6 H7 and m^1^A4 H8-T2 H2′/H2′′ further supported the location of T2 and T6 in the minor groove (Figure S5D). In the m^1^A MDB, T6 was closer to the loop-closing base pairs than T2, allowing the formation of hydrophobic contacts between the methyl group of T6 and 2′-methylene groups of m^1^A4 and T5 (Figure 3E). In addition, T6 H3 formed a hydrogen bond with T1 O2 (Figure 3E), with a bond distance and angle of ≈1.86 Å and 154°, respectively. T2 partially stacked on T6, and its methyl group was in hydrophobic contact with the 2′-methylene group of T1 (Figure 3F). In three out of five refined structures, T2 O4 showed Na^+^-mediated electrostatic interactions with O4′ and OP1 of T6 (Figure S6).

T3 and T7 stacked on the T1·m^1^A4 and T5-A8 base pairs, respectively (Figure 3G), as suggested by the NOEs of T3 H6-m^1^A4 H8, T3 H7-T1 H6, T7 H6-A8 H2, and T7 H7-T5 H6 (Figure S5A,E). The upfield shifted signals of T3 and T7 H7/H1′ (1.53/5.24 ppm for T3, and 1.61/5.58 ppm for T7) (Figure 2A and Figure S1) were attributed to shielding effects from the T1·m^1^A4 and T5-A8 base pairs, respectively. The methyl groups of T3 and T7 formed hydrophobic contacts with 2′-methylene groups of T1/T2 and T5/T6, respectively (Figure 3H).

By comparing the high-resolution structure of the unmodified TTTA MDB (Figure 1C, PDB ID: 5GWQ) with that of the m^1^A MDB, two obvious structural differences upon m^1^A modification were observed. Firstly, T1-A4 in the TTTA MDB adopted a Watson–Crick pairing mode, whereas T1·m^1^A4 in the m^1^A MDB adopted a Hoogsteen pairing mode as the methyl group of m^1^A impeded the formation of a Watson–Crick base pair (Figure S7) [17,20]. The different base pairing mode led to shortening of the C1′-C1′ distance (the observed T1 C1′-m^1^A4 C1′ distance of 9.1 ± 0.2 Å in the m^1^A MDB versus the T1 C1′-A4 C1′ distance of 10.4 ± 0.2 Å in the TTTA MDB). It has been suggested that a shorter C1′-C1′ distance of the loop-closing base pair would favor the stacking with L3 residue in a type II tetraloop [29]. Secondly, T2 was found to be closer to the loop-closing base pairs due to stabilization of a hydrogen bond with T5 or T7 and hydrophobic contacts with T1 and A8 in the TTTA MDB [23], whilst T6 was closer to loop-closing base pairs as stabilized by the formation of a hydrogen bond with T1 and hydrophobic contacts with m^1^A4 and T5 in the m^1^A MDB (Figure 3E). Yet, such stabilizing forces in these two MDBs seemed to be comparable and should not be the main factor that determined the relative positions of T2 and T6. Therefore, the inner position of T6 in the m^1^A MDB is likely attributed to the more stable T5-A8 Watson–Crick base pair than the T1·m^1^A4 Hoogsteen base pair, which may lead to a prior folding of the 3′-loop followed by the folding of the 5′-loop with T2 capping on T6.

### 2.2. Conformational Exchange between Two MDB Structures in 5′-TTTm^6^ATTTA-3′

**Figure 4:**
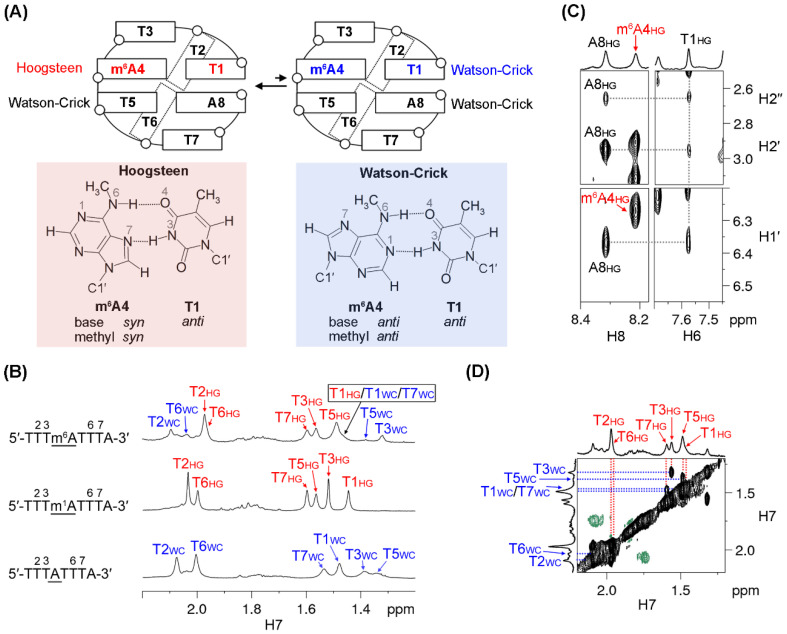
(**A**) 5′-TTTm^6^ATTTA-3′ displayed a conformational exchange between two MDBs with T1·m^6^A4 Hoogsteen and Watson–Crick base pairs, respectively. (**B**) Comparison of the H7 signals of 5′-TTTm^6^ATTTA-3′ (top) with those in the m^1^A MDB (middle) and the TTTA MDB (bottom). The 1D ^1^H spectra were acquired at 0 °C. The subscripts “WC” and “HG” mean that T1·m^6^A4/ T1·m^1^A4/T1-A4 formed a Watson–Crick and Hoogsteen base pair respectively, in the corresponding structure. (**C**) NOEs of A8 H1′/H2′/H2′′-T1 H6 supported the formation of an MDB for the major conformer. The NOESY spectrum was acquired at 15 °C with a mixing time of 600 ms. (**D**) The H7 signals of the minor conformer were assigned based on their exchange cross peaks with the corresponding signals of the major conformer in a rotating frame Overhauser effect spectroscopy (ROESY) spectrum. The ROESY spectrum was acquired at 0 °C with a spinlock time of 200 ms.

The m^6^A-modified 5′-TTTm^6^ATTTA-3′ sequence was found to exhibit two MDB conformers, namely a major and a minor conformer, which underwent a slow conformational exchange at low temperatures. In the major MDB conformer, T1·m^6^A4 formed a Hoogsteen base pair in which both the base and *N^6^*-methyl of m^6^A4 exhibited a *syn* orientation. On the other hand, T1·m^6^A4 in the minor MDB conformer formed a Watson–Crick base pair with m^6^A adopting *anti* base and *anti N^6^*-methyl orientations (Figure 4A).

A 1D ^1^H NMR spectrum of 5′-TTTm^6^ATTTA-3′ was acquired at 0 °C, which showed two sets of ^1^H signals (Figure 4B and Figure S8A). When the temperature was raised to 15 °C, the signals of the minor conformer almost vanished. This allowed us to conduct sequential resonance assignments (Figure S2) and structural analysis for the major conformer at 15 °C. Notably, the major conformer adopted an MDB structure containing two type II tetraloops, as suggested by (i) the downfield shifted T2 and T6 H6/H7 signals (7.72/1.91 ppm for T2, and 7.69/1.89 ppm for T6), and (ii) the upfield shifted T3/T7 H7 signals (1.66/1.68 ppm) (Figure 4B and Figure S8A). The NOEs of A8 H1′/H2′/H2′′-T1 H6 (Figure 4C) also suggested a close proximity between the terminal residues A8 and T1, and thus further corroborated the formation of the MDB structure. Unfortunately, signals of T1 and T5 H3 were not observed probably due to a rapid exchange with water at 15 °C. This rendered the analysis of base pairing modes difficult to carry out. Alternatively, it was found that (i) the NOE of m^6^A4 H8-m^6^A4 H1′ was stronger than the NOE of m^6^A4 H8-m^6^A4 H2′, and (ii) the NOE of m^6^A4 H8-H1′ appeared to be stronger than the intra-nucleotide NOE of H6/H8-H1′ of other residues (Figure 4C and Figure S2 and Table S3). These suggested that m^6^A4 adopted a *syn* base orientation [30,34] and thus T1·m^6^A4 formed a Hoogsteen base pair of the major MDB conformer. In addition, the chemical shifts of all methyl signals in the major conformer were similar to those observed for the m^1^A MDB (Figure 4B). This is also in good agreement with the T1·m^6^A4 Hoogsteen and the T5-A8 Watson–Crick base pairing modes.

The attempt to determine the thermodynamic stability of this major m^6^A MDB conformer via UV melting experiments was unsuccessful, as both the major and minor conformers contributed to the UV absorbances at low temperatures, which would bring about uncertainties to the thermodynamic parameters obtained from curving fitting using the two-state transition model [35]. Alternatively, we performed 1D ^1^H NMR melting experiments to monitor the chemical shift changes of T2, T5, and T6 H7 signals of the major m^6^A MDB conformer as a function of temperature (Figure S9). The *T_m_* value of this major MDB conformer was estimated to be ≈10 °C, which was much lower than that of the unmodified TTTA MDB. Such low thermal stability also hindered the structural calculation of this major MDB conformer, as the NMR signals reflected a weighted average of the folded and unfolded structures.

As the NMR signals of the minor conformer were too weak to conduct sequential resonance assignments using a NOESY spectrum, we made use of a rotating frame Overhauser effect spectroscopy (ROESY) spectrum to carry out ^1^H signal assignments for the minor conformer (Figure 4D and Figure S8B). It is worth noting that (i) the downfield shifted T2 and T6 H6/H7 signals (7.91/2.10 ppm for T2, and 7.85/2.04 ppm for T6) and (ii) the upfield shifted T3/T7 H7 signals (1.32/1.48 ppm) (Figure 4B and Figure S8) support that the minor conformer was also an MDB structure. The chemical shifts of thymine methyl protons in the minor conformer were found to map well with those of the corresponding thymine methyl protons in the TTTA MDB, which contained two T-A Watson–Crick loop-closing base pairs (Figure 4B). Therefore, it is reasonable to envisage that both T1·m^6^A4 and T5-A8 formed Watson–Crick base pairs in this minor MDB structure. In addition, the minor MDB conformer would unfold first when the temperature was raised (Figure S8A), suggesting that it had a lower thermodynamic stability than the major MDB conformer. This also agreed with our observations that the MDB with a T1-A4 Watson–Crick base pair was less stable than the one with a T1·m^1^A4 Hoogsteen base pair (Table 1). The populations of the major and minor m^6^A MDB conformers were estimated to be ≈67% and ≈33%, respectively. This estimation was based on the intensities of their respective T3 H7 signal, which was well resolved at 0 °C (Figure 4B).

## 3. Discussion

NMR structural analyses revealed that 5′-TTTm^1^ATTTA-3′ folded into an MDB with a T1·m^1^A4 Hoogsteen base pair. The formation of the T1·m^1^A4 Hoogsteen base pair is consistent with those observed in DNA duplex [17,20]. It is remarkable to note that a T·m^1^A Hoogsteen base pair brought about a higher thermodynamic stability to MDB as compared to an unmodified T-A Watson–Crick base pair. This finding is different from the reported destabilization effect of m^1^A on DNA duplex [15]. To the best of our knowledge, this is the first report on the stabilization effect of m^1^A on a DNA secondary structure. In addition, we previously reported that the inner position of T2 rather than T6 in the minor groove of the TTTA MDB could lead to a translational motion of the MDB from 5′ to 3′ direction in a long tract of repeats [36]. In contrast, m^1^A modification leads to the formation of an MDB with T6 rather than T2 at the inner position of the minor groove; one would expect to observe an opposite translational motion (from 3′ to 5′ direction) of the MDB in a long repeating sequence upon m^1^A modification. Further studies can be conducted to investigate how adenine methylation affects structural dynamics of MDB in longer repeats, and this may benefit us in understanding the effects of adenine methylation on non-B DNA structure-mediated genetic instabilities.

The structural behavior of the 5′-TTTm^6^ATTTA-3′ sequence is a bit more complicated than that of 5′-TTTm^1^ATTTA-3′. The 5′-TTTm^6^ATTTA-3′ sequence adopted a major MDB conformer containing a T1·m^6^A4 Hoogsteen base pair (with *syn N^6^*-methyl in m^6^A4) as well as a minor MDB conformer containing a T1·m^6^A4 Watson–Crick base pair (with *anti N^6^*-methyl in m^6^A4) with a population ratio of approximately 2:1 at 0 °C. This ratio is significantly different from the ratios of ≈20:1 and 0:1 reported for *syn*:*anti N^6^*-methyl conformations of m^6^A in the unpaired base [21] and RNA duplex [22], respectively, suggesting that the *N^6^*-methyl conformation of m^6^A may depend largely on the corresponding structural environments. Based on the NMR melting profiles, the *T_m_* of the major m^6^A MDB conformer was estimated to be ≈10 °C. The *T_m_* of the minor m^6^A MDB conformer was even lower as it unfolded prior to the unfolding of the major conformer. Comparing with the *T_m_* of the unmodified TTTA MDB, m^6^A modification leads to destabilization in the MDB structure, which is consistent with the reported destabilization effects on duplex containing m^6^A·T base pairs [13,21]. On the other hand, it has been reported that unpaired m^6^A had stabilization effects on duplex and G-quadruplex structures due to improved stacking interactions [19,22]. Therefore, one can deduce that the destabilizing effect upon substituting an T-A Watson–Crick base pair by an T·m^6^A base pair outweighed the improved stacking interactions resulting from m^6^A.

## 4. Materials and Methods 

### 4.1. DNA Samples

The 5′-TTTm^1^ATTTA-3′ sequence was synthesized by an Applied Biosystems model 394 DNA/RNA synthesizer (Foster City, CA, USA). The sample was first deprotected in a 30% ammonium hydroxide solution at 35 °C for 18 h to minimize the occurrence of Dimroth rearrangement [12,14]; then, it was purified by denature polyacrylamide gel electrophoresis and diethylaminoethyl Sephacel anion exchange chromatography, and finally desalted by Amicon Ultra-4 centrifugal filtering devices. The 5′-TTTm^6^ATTTA-3′ sequence was purchased from Sangon Biotech Co. Ltd. (Shanghai, China) and further purified by diethylaminoethyl Sephacel anion exchange chromatography followed by desalting. NMR samples were prepared by dissolving 0.15–0.25 μmol purified DNA into 500 μL buffer solutions containing 0.02 mM 2,2-dimethyl-2-silapentane-5-sulfonate (DSS) and 10 mM sodium phosphate (NaPi) at pH 7 in 90% H_2_O/10% D_2_O or 99.96% D_2_O.

### 4.2. NMR Experiments

NMR experiments were conducted using a Bruker AVANCE 700 MHz, 600 MHz or 500 MHz spectrometer (Billerica, MA, USA). For studying labile protons, the DNA samples were prepared in 90% H_2_O/10% D_2_O, and jump-return [37] or excitation sculpting [38] pulse sequences were employed in 1D ^1^H, 2D NOESY, or 2D ROESY experiments to suppress the water signal. For studying nonlabile protons, the samples were prepared in 99.96% D_2_O, and a 2-second presaturation pulse was applied to suppress the residual HDO signal. Two-dimensional (2D) NOESY and TOCSY spectra were acquired with datasets of 4096 × 512 and zero-filled to produce 4096 × 4096 spectra with a cosine window function applied to both dimensions. The 2D ROESY spectrum was acquired with a dataset of 4096 × 256 and zero-filled to produce 4096 × 4096 spectra with a cosine window function. Two-dimensional (2D) double-quantum-filtered correlation spectroscopy (DQF-COSY) was acquired with a dataset of 4096 × 512 and zero-filled to give 4096 × 4096 with a sine window function. In a typical ^1^H-^31^P HSQC experiment, a Carr–Purcell–Meiboom–Gill pulse train [39] with surrounding delays of ≈100 μs was applied during magnetization transfer between phosphorus and proton. In a typical ^1^H-^13^C HMBC experiment, a delay of 65 ms was used for the evolution of long-range couplings [28]. In the ^1^H-^13^C HSQC experiment, the coupling constant was set at 180 Hz for studying carbons in nucleobases. For ^1^H-^31^P HSQC, ^1^H-^13^C HMBC, and ^1^H-^13^C HSQC experiments, datasets of 4096 × 200 were acquired and zero-filled to give 4096 × 2048 spectra. ^31^P and ^13^C chemical shifts were indirectly referenced to DSS using the derived nucleus-specific ratios of 0.404808636 and 0.251449530, respectively [30].

To investigate the thermal stability of the major MDB conformer in 5′-TTTm^6^ATTTA-3′, we performed NMR melting experiments by acquiring 1D ^1^H spectra from 0 to 60 °C with a step size of 2.5 °C per spectrum. The NMR melting curves were constructed by plotting the chemical shifts of T2, T5, and T6 H7 signals as a function of temperature. The *T_m_* values were determined by fitting the melting curves using the two-state transition model [35].

### 4.3. Resonance Assignments

Based on the assignments of H6/H8/H1′ signals using standard methods [27], H2′/H2′′ were assigned using H2′/H2′′-H1′ correlations in the DQF-COSY spectrum and distinguished by the stronger H2′′-H1′ NOE than H2′-H1′ NOE [34]. Upon assigning H3′ signals based on H1′/H2′/H2′′-H3′ correlations in the TOCSY spectrum, H4′ signals were assigned based on H3′-H4′ correlations in the DQF-COSY spectrum. H5′/H5′′ signals were assigned by analyzing intensities of intra-nucleotide H3′-H5′/H5′′ and H4′-H5′/H5′′ NOEs and comparing the corresponding ^3^*J*_H4′H5′/H5′′_ coupling constants [27]. ^1^H and ^31^P chemical shifts of m^1^A MDB are shown in Table S1.

### 4.4. Extraction of Experimental NMR Restraints

Solution structures of the m^1^A MDB were calculated with experimental NMR restraints. NOE-derived distance restraints were extracted from the NOESY spectrum acquired at 5 °C with a mixing time of 600 ms. Based on their relative intensities, NOEs were classified into strong, strong to medium, medium, medium to weak, and weak groups, and distance ranges of 1.8–4.0, 2.5–4.5, 3.0–5.0, 3.5–5.5, and 4.0–6.0 Å were applied, respectively. A distance rage of 1.8–5.0 Å was employed for NOEs involving labile protons, and a range of 1.8–6.0 Å was given to NOEs with signal overlapping or broadening. Herein, we used relatively loose distance restraint ranges to avoid an overestimation on the calculated structures due to tight restraints. After determining base pairing modes, hydrogen bond restraints were applied to the T1·m^1^A4 Hoogsteen base pair [16,40] and T5-A8 Watson–Crick base pairs [40]. Backbone torsion angle *γ* was determined by analyzing the intensities of intra-nucleotide H3′-H5′/H5′′ and H4′-H5′/H5′′ NOEs and comparing the corresponding ^3^*J*_H4′H5′/H5′′_ coupling constants [27]. Glycosidic torsion angle χ was determined by comparing relative intensities of H6/H8-H2′ and H6/H8-H1′ NOEs [30]. For m^1^A4 in *syn* base orientation, a glycosidic torsion angle range of 21–101° was applied [17]. The H1′-C1′-C2′-H2′ dihedral angles of the deoxyribose were calculated based on ^3^*J*_H1′H2′_ coupling constants using Karplus equation [41] with a freedom of ± 10°. Chirality restraints were directly generated by AMBER [42] in the form of improper torsions. The same chirality restraints were manually added to m^1^A4. Restraints used in the calculation of the m^1^A4 MDB were summarized in Table S2.

### 4.5. rMD–rEM Calculations

Calculations of the m^1^A MDB were performed on AMBER 16 (San Francisco, CA, USA) with the OL15 force field [42,43]. Force field parameters of the m^1^A4 residue were generated by using the adenine as an initial template. Upon adding a methyl group (the carbon and three hydrogen atoms were named as CN, HN1, HN2, and HN3, respectively) to the N1 atom, the bond lengths of N1-CN and CN-HN1/2/3 were set to be 1.47 and 1.09 Å, respectively, and the angles of HN1-CN-HN2, HN1-CN-HN3, and the HN2-CN-HN3 were all set to be 109.5°. Partial charges of the m^1^A4 residue were adopted from reference [44]. To obtain the initial structure of single-stranded 5′-TTTm^1^ATTTA-3′, we first built a 5′-TTTATTTA-3′/5′-TAAATAAA-3′ B-DNA using Nucleic Acid Builder, which was followed by removal of the 5′-TAAATAAA-3′ strand and substitution of A4 with m^1^A4. Owing to the presence of a positive charge on the N1 atom of the m^1^A4 residue, six Na^+^ ions were added to neutralize negative charges on the backbone. Then, the system was energy minimized.

The in vacuo rMD calculations were initiated by heating the system from 300 to 1000 K in 5 ps, during which the weighting of restraints was increased from 0.1 to 5.0. Then, the system was maintained at 1000 K for 10 ps, prior to slowly cooling to 300 K in 25 ps. The restraint weighting was maintained at 5.0 at 1000 K and then gradually reduced to 1.0 during the cooling process. Finally, the system underwent an equilibration period of 5 ps at 300 K with a restraint weighting of 1.0. Potentials of NOE-based distance restraints, hydrogen bond restraints, chirality, backbone, and the H1′-C1′-C2′-H2′ torsion angles of the deoxyribose were 1000, 500 kcal·mol^−1^·Å^−2^, 500, 500, and 500 kcal·mol^−1^·rad^−2^, respectively. Relatively high restraint potentials were used as smaller values such as 20 kcal·mol^−1^·Å^−2^ or kcal·mol^−1^·rad^−2^ lead to misfolded structures with significant restraint violations and deviations from ideal geometries in our trials. The resulting structures at the end of rMD were subjected to rEM by 200 steps of steepest descent, and then the conjugate gradient until the energy gradient difference between successive minimization steps was smaller than 0.1 kcal·mol^−1^·Å^−2^. A total of 100 independent rMD-rEM experiments were performed with random velocities (random seeds), and five structures with the lowest restraint violation energies were selected as a final representative ensemble (PDB ID: 7E4E).

### 4.6. Analyses of Calculated Structures

The calculated structures were analyzed using CPPTRAJ [45] and suppose modules of AMBER [42]. UCSF Chimera (San Francisco, CA, USA) [46] was used for structural visualization and plotting.

### 4.7. UV Melting Experiments

UV melting experiments were carried out to determine the thermodynamic stability of the m^1^A MDB. UV melting samples were prepared by dissolving 5 μM DNA in 1000 μL 10 mM NaPi at pH 7 and then transferred to a 1 cm cuvette. Absorbances at 260 nm were collected as a function of temperature by a Hewlett-Packard 8453 diode-array UV-visible spectrophotometer (Agilent Technologies, Santa Clara, CA, USA), with solution temperature monitored by a BetaTHERM thermistor temperature sensor inserted into the cuvette. Absorbances were measured from 10 to 65 °C with a step size of 1 °C and hold time of 1 min. A nitrogen gas purge was used at temperature below 18 °C to prevent water condensation on the cuvette. Three replicative measurements were conducted, and the melting profiles were fitted with a two-state transition model [35] for the elucidation of thermodynamic parameters. Additionally, we ran a heat-and-cool cycle, and we found that the heating and cooling profiles overlapped well (Figure S10), suggesting that (i) the folding and unfolding processes appeared reversible under the temperature gradient we used, and (ii) the m^1^A residue did not convert to m^6^A at high temperatures.

## Figures and Tables

**Table 1:** Thermodynamic parameters *^a^* of the m^1^A MDB and TTTA MDB.

MDB	Sequence	*T_m_* (°C)	∆*H°* (kcal·mol^−1^)	∆*S°* (cal·K^−1^·mol^−1^)	∆*G°_25 °C_* (kcal·mol^−1^)
m^1^A	5′-TTTm^1^ATTTA-3′	23.2 ± 0.6	−19 ± 2	−64 ± 6	0.12 ± 0.04
TTTA *^b^*	5′-TTTATTTA-3′	19.2 ± 0.9	−22 ± 1	−76 ± 4	0.43 ± 0.08

*^a^*Average values and uncertainties were obtained by three replicative measurements. *^b^*Data for the TTTA MDB were extracted from reference [32].

**Table 2:** NMR refinement statistics of the m^1^A MDB.

Restraint Satisfaction	
Number of distance restraint violation > 0.2 Å	0
Number of angle restraint violation > 5°	0
Deviation from ideal geometry	
Bond distance (Å)	0.0097 ± 0.0002
Angle (°)	2.69 ± 0.08
Heavy atomic RMSD (Å) *^a^*	
Average pairwise RMSD	0.62 ± 0.09
RMSD from mean structure	0.39 ± 0.07

*^a^* RMSD values were calculated for the five representative structures.

## Data Availability

NMR solution structures of the m^1^A MDB have been deposited to Protein Data Bank under accession number 7E4E.

## Supplementary Materials

The following are available online at https://www.mdpi.com/article/10.3390/ijms22073633/s1, Table S1: ^1^H and ^31^P chemical shifts of the m^1^A MDB, Table S2: Experimental NMR restraints used in structural calculation of the m^1^A MDB, Table S3: Comparison of relative intensities of intra-nucleotide H6/H8-H1′ and H6/H8-H2′ NOEs in 5′-TTTm^6^ATTTA-3′, Figure S1: Sequential resonance assignment of the m^1^A MDB, Figure S2: Sequential resonance assignment of 5′-TTTm^6^ATTTA-3′, Figure S3: ^31^P resonance assignment of the m^1^A MDB, Figure S4: Adenine H2 assignment of the m^1^A MDB, Figure S5: NOEs supporting structural features of the m^1^A MDB, Figure S6: m^1^A MDBs with electrostatic interactions between T2 and T6, Figure S7: Schematic of T·m^1^A base pair, Figure S8: Variable-temperature 1D ^1^H spectra and 2D ROESY spectrum of 5′-TTTm^6^ATTTA-3′, Figure S9: NMR melting curves of 5′-TTTm^6^ATTTA-3′ major conformer, Figure S10: UV heating and cooling profiles of the m^1^A MDB.

## Abbreviations

m^1^A	*N^1^*-methyladenine
m^6^A	*N^6^*-methyladenine
MDB	minidumbbell
*T_m_*	melting temperature
NMR	nuclear magnetic resonance
NOESY	nuclear Overhauser effect spectroscopy
HSQC	heteronuclear single-quantum correlation
TOCSY	total correlation spectroscopy
HMBC	heteronuclear multiple bond correlation
rMD	restrained molecular dynamics
rEM	restrained energy minimization
UV	ultraviolet
ROESY	rotating frame Overhauser effect spectroscopy
DSS	2,2-dimethyl-2-silapentane-5-sulfonate
NaPi	sodium phosphate
DQF-COSY	double-quantum-filtered correlation spectroscopy
