# Supplementary material for: Effects of Adenine Methylation on the Structure and Thermodynamic Stability of a DNA Minidumbbell
Source: Int J Mol Sci. 2021 Mar 31;22(7):3633. doi: 10.3390/ijms22073633 (PMC8037738; doi:10.3390/ijms22073633)

Supporting Information

Effects of Adenine Methylation on the Structure and Thermodynamic Stability of a DNA Minidumbbell

Liqi Wan [2], Sik Lok Lam [2,*], Hung Kay Lee [2] and Pei Guo [1,*]

[1] School of Biology and Biological Engineering, South China University of Technology, Guangzhou, Guangdong 510006, China.
[2] Department of Chemistry, The Chinese University of Hong Kong, Shatin, New Territories, Hong Kong SAR, China.
* Correspondence: peiguo@scut.edu.cn, lams@cuhk.edu.hk

Table of Contents

Tables S1-S3 (Page 2-4);

Figures S1-S10 (Page 5-14).

Table S1. ^{1}H and ^{31}P chemical shifts (ppm) of the m^{1}A MDB[a].

	H2/H7	H3/ N^{1}Me	H61/H62	H6/H8	H1′	H2′	H2″	H3′	H4′	H5′	H5″	^{31}P
T1	1.46	12.37	-	7.61	6.19	2.07	2.44	4.89	4.39	3.91	3.91	-
T2	2.03	[b]	-	7.90	6.42	2.11	2.49	4.86	4.43	4.25	4.14	-3.11
T3	1.53	[b]	-	7.59	5.24	1.81	2.05	4.64	4.09	3.87	4.00	-5.08
m^{1}A4[c]	8.62	4.01	9.28/9.62	8.37	6.36	3.35	3.05	5.05	4.48	4.11	4.23	-4.27
T5	1.58	13.87	-	7.58	6.29	2.33	2.38	4.84	4.51	4.31	4.02	-4.18
T6	2.00	[b]	-	7.83	6.34	2.10	2.46	4.80	4.40	4.26	4.13	-3.48
T7	1.61	[b]	-	7.54	5.58	1.77	2.05	4.57	3.34	3.80	3.88	-4.91
A8	8.18	-	[d]	8.29	6.33	3.05	2.72	4.86	4.19	3.94	3.91	-3.98

[a] Chemical shifts of labile and nonlabile protons were measured at 0 and 5 °C, respectively.

[b] Chemical shift could not be measured due to signal overlapping.

[c] For m^{1}A4, the protons of methyl group attached to N1 are named as N^{1}Me.

[d] Chemical shift could not be measured due to signal broadening.

Table S2. Experimental NMR restraints used in structural calculation of the m¹A MDB.

Distribution of NOE-derived distance restraints (intra-nucleotide/inter-nucleotide = 168/165)

	T1	T2	T3	m¹A4	T5	T6	T7	A8
T1	12	16	6	1				15
T2		24	19	4		2		2
T3			18	9				
m¹A4				29	20	5		1
T5					22	22	9	1
T6						24	21	2
T7							22	10
A8								17

Hydrogen bond restraints

Atom pair	Distance restraint (Å)
T1 O4-m¹A4 N6	2.76-3.20
T1 N3-m¹A4 N7	2.83-3.30
T5 O4-A8 N6	2.85-3.05
T5 N3-A8 N1	2.72-2.92

Deoxyribose, backbone (γ) and glycosidic (χ) torsion angle restraints

Residue	$^3J_{H1'H2'}$ (Hz)	H1′-C1′-C2′-H2′ dihedral angle (°)	$^3J_{H4'H5'}$ (Hz)	$^3J_{H4'H5''}$ (Hz)	γ (°)	χ (°)
T1	9.4	147-167	[a]	[a]	-	90-270
T2	9.2	146-166	[a]	[b]	30-90 (*gauche*+)	90-270
T3	10.1	153-173	[b]	[a]	30-90 (*gauche*+)	90-270
m¹A4	[c]	-	[c]	[c]	150-210 (*trans*)	21-101
T5	10.1	153-173	4.0	8.4	150-210 (*trans*)	90-270
T6	9.4	147-167	[a]	[b]	30-90 (*gauche*+)	90-270
T7	[c]	-	[b,c]	[b,c]	30-90 (*gauche*+)	90-270
A8	4.0	116-136	[b]	[c]	150-210 (*trans*)	90-330

[a] The coupling constant could not be measured due to peak overlapping.
[b] The coupling constant could not be measured due to weak coupling.
[c] The coupling constant could not be measured due to peak broadening.

Chirality restraints for all residues

C2′, O4′, N1/N9, H1′	C3′, C1′, H2′, H2″	O3′, C2′, C4′, H3′	C3′, C5′, O4′, H4′	C4′, O5′, H5′, H5″
60-80°	60-80°	60-80°	60-80°	60-80°

Table S3. Relative intensities of intra-nucleotide H6/H8-H1′ and H6/H8-H2′ NOEs in 5′-TTTm⁶ATTTA-3′.[a]

Residue	H6/H8-H1′ NOE	H6/H8-H2′ NOE
T1	32.91	137.97
T2	[b]	[b]
T3	51.19	[b]
m¹A4	100.00	85.54
T5	39.28	[b]
T6	[b]	[b]
T7	34.08	[b]
A8	51.67	76.64

[a] The NOE cross peaks were integrated from NOESY spectrum acquired at 15 °C with a mixing time of 600 ms.

[b] The NOE intensity could not be measured due to peak overlapping.

Figure S1. Sequential resonance assignment of the m^{1}A MDB using the NOESY H6/H8-H1′ fingerprint region. The spectrum was acquired at 5 °C with a mixing time of 600 ms.

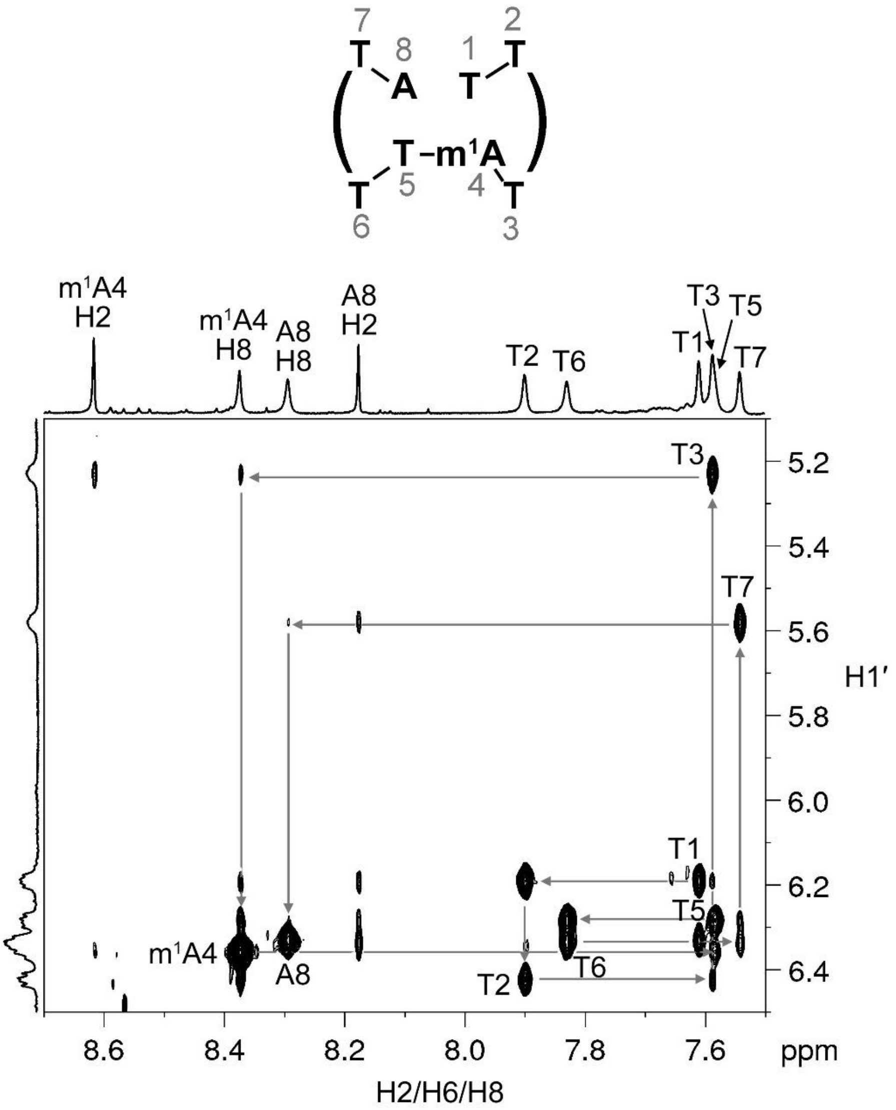

Figure S2. Sequential resonance assignment of 5′-TTT<u>m⁶A</u>TTTA-3′ using the NOESY H6/H8-H1′/H2′/H2″ fingerprint regions. m^6A4 exhibited a stronger intra-nucleotide H6/H8-H1′ NOE than the other residues. The spectrum was acquired at 15 °C with a mixing time of 600 ms.

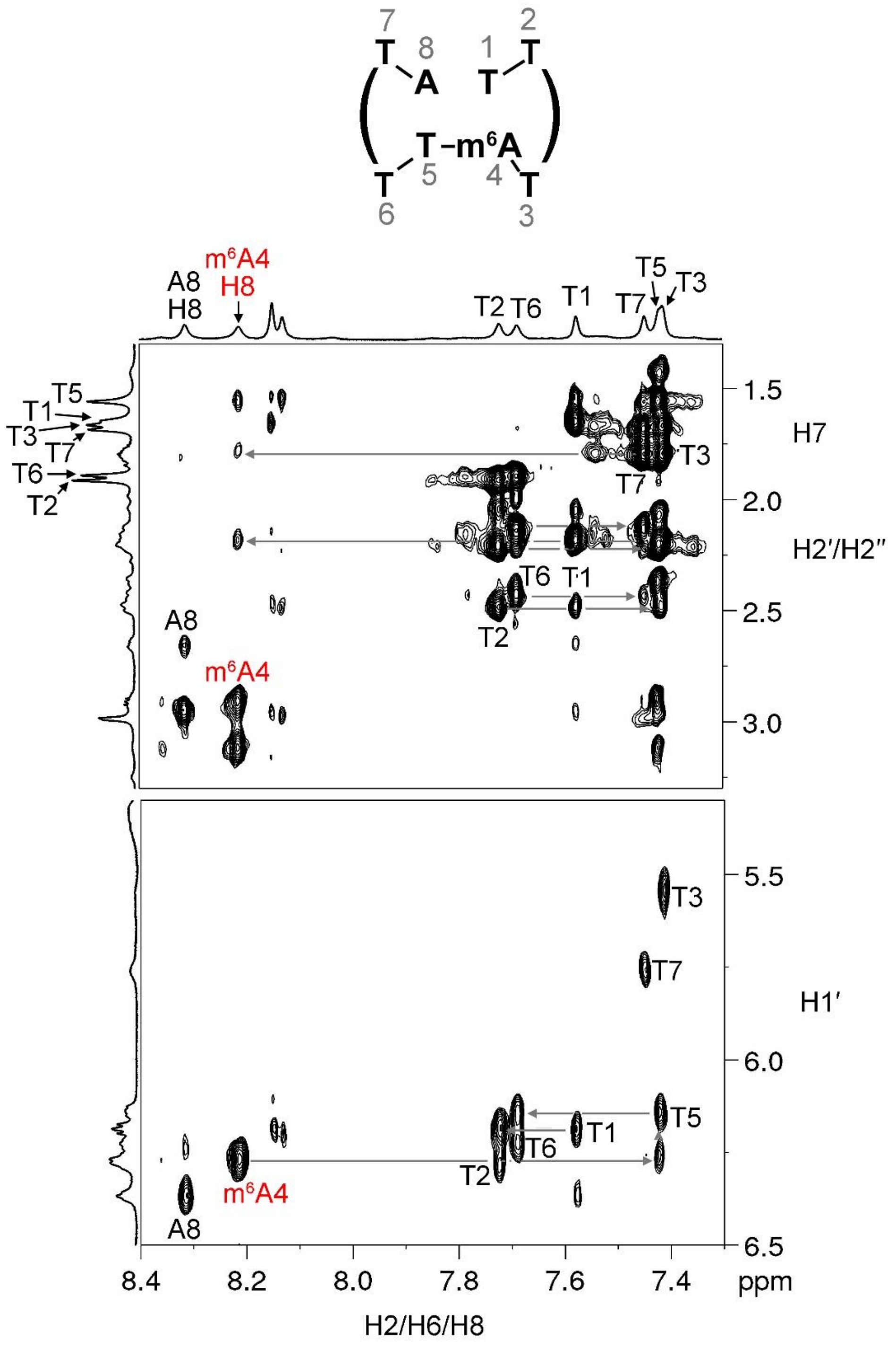

Figure S3. ^{31}P resonance assignment of the m^{1}A MDB using intra-nucleotide H2′/H2″-H3′ and H1′-H3′ TOCSY cross peaks (top and middle) and inter-nucleotide H3′-^{31}P HSQC cross peaks (bottom). The spectra shown here were acquired at 5 °C.

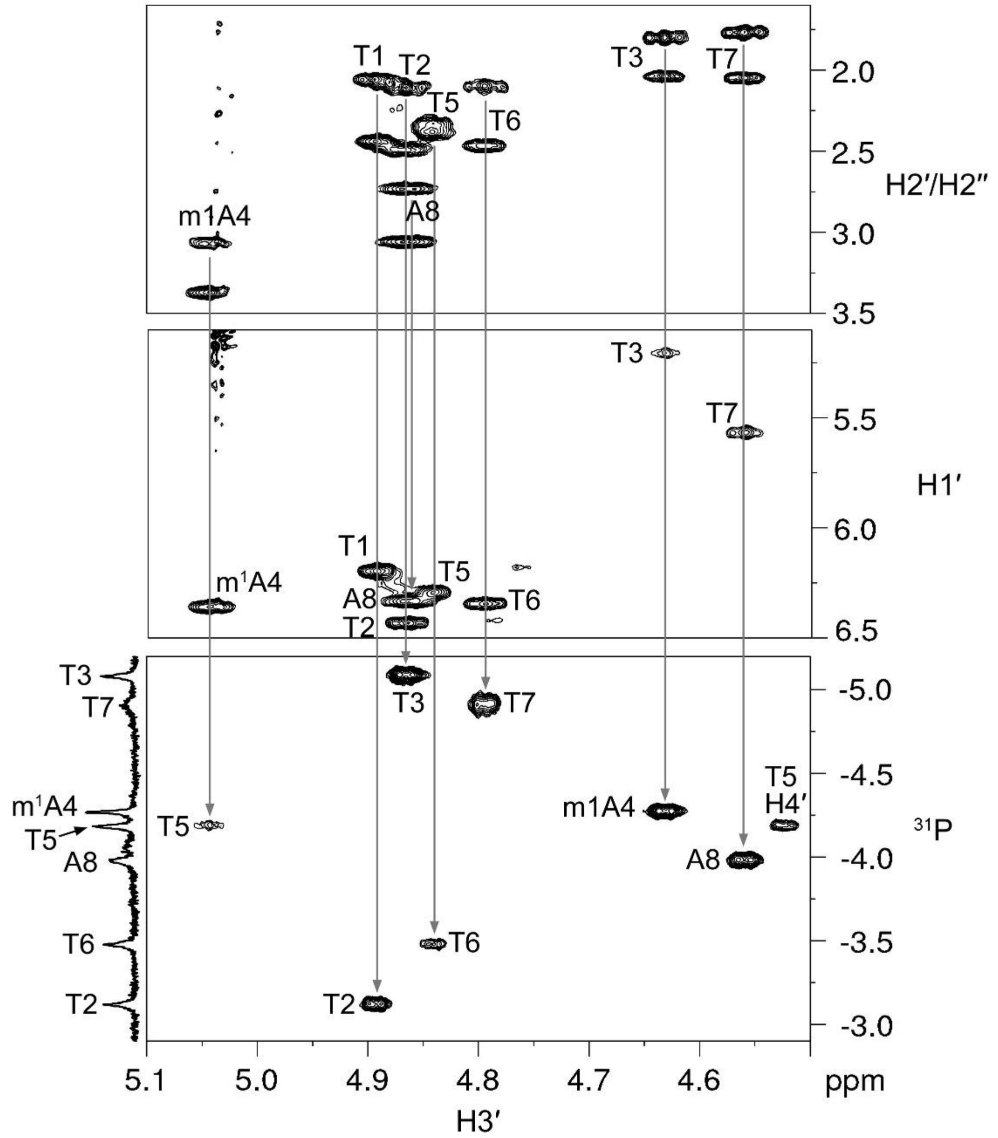

Figure S4. Adenine H2 resonance assignment of the m[1]A MDB using long-range H2-C4 and H8-C4 correlations in the HMBC spectrum. The spectrum shown here was acquired at 5 °C.

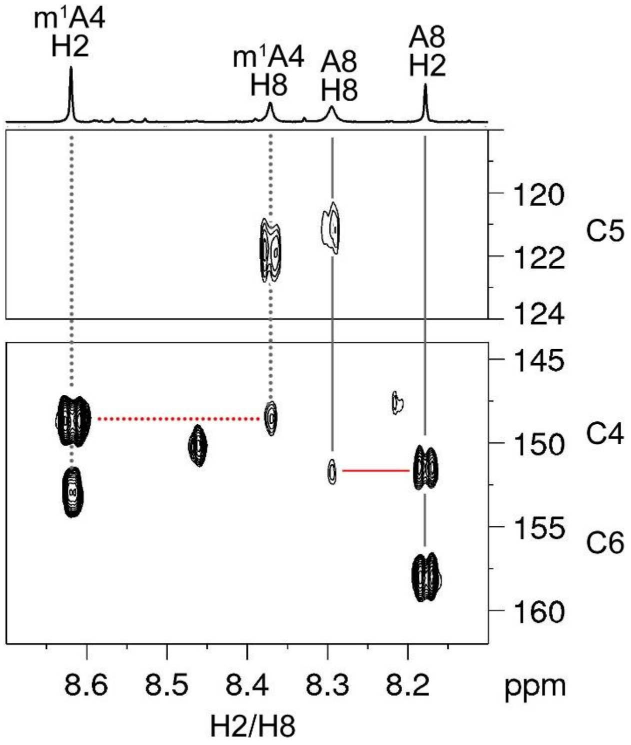

Figure S5. In the m¹A MDB, (A) the NOEs of T1 H6-A8 H8 and T5 H6-m¹A4 H8 supported the base-base stacking between T1·m¹A4 and T5-A8 base pairs, and the NOEs of T3 H6-m¹A4 H8 and T7 H6-A8 H2 supported that T3 and T7 stacked on m¹A4 and A8, respectively. (B) The NOE of m¹A4 H8-A8 H2 agreed with the stacking between T1·m¹A4 and T5-A8 base pairs. (C) The NOEs of A8 H2-T2 H7 and A8 H2-T6 H2′/H2″, and (D) m¹A4 H8-T2 H2′/H2″ and m¹A4 H8-T6 H7 supported that T2 and T6 were located in the minor groove. (E) The NOEs of T3 H7-T1 H6 and T7 H7-T5 H6 supported that T3 and T7 stacked on T1 and T5, respectively. The spectra shown here were acquired at 5 °C with a mixing time of 450 ms.

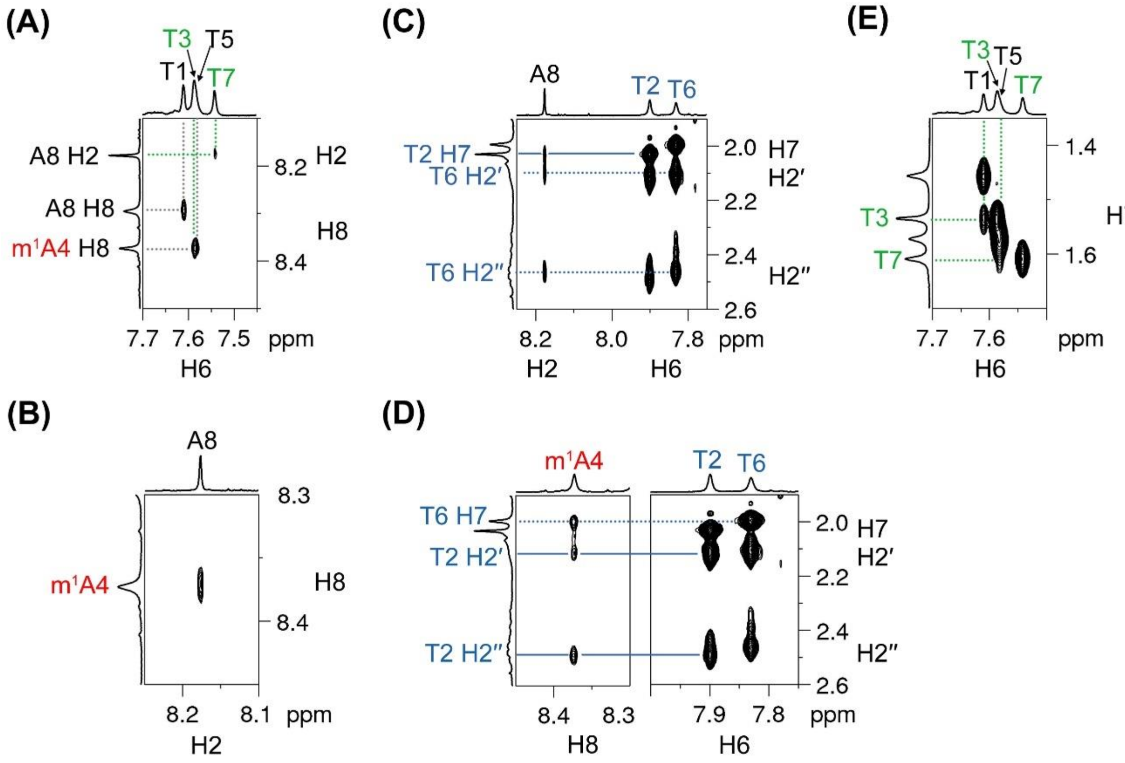

Figure S6. In the NMR solution structures of the m^{1}A MDB (PDB ID: 7E4E), three out of five showed Na^+-mediated electrostatic interactions among T2 O4, T6 O4′ and T6 OP1.

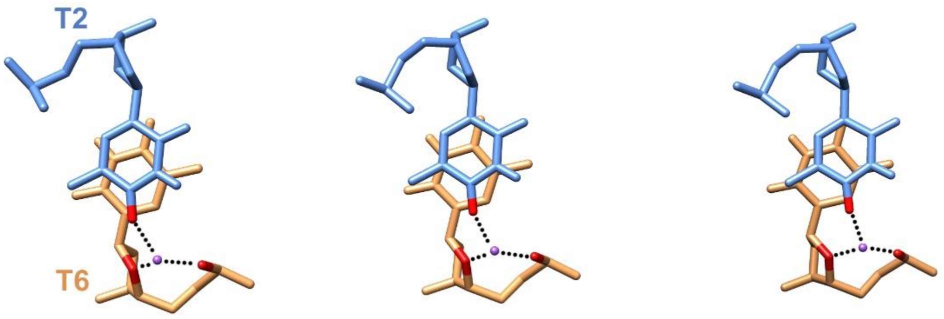

Figure S7. A schematic showing that the presence of a methyl group in m¹A impedes the formation of a Watson-Crick base pair.

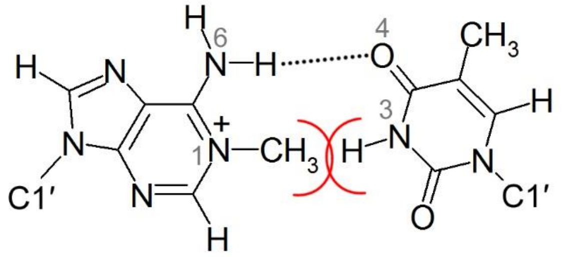

N¹-methyladenine (*anti*) thymine (*anti*)

Figure S8. (A) Variable-temperature 1D ^{1}H spectra of 5′-TTTm^{6}ATTTA-3′ showing two sets of signals. Signals of the major conformer (an MDB containing T1·m^{6}A4 Hoogsteen base pair) and the minor conformer (an MDB containing T1·m^{6}A4 Watson-Crick base pair) are labeled in red with a subscript of HG and blue with a subscript of WC, respectively. At elevated temperatures, signals of the minor conformer are briefly labeled by "*". (B) H6/H8 signals in the minor conformer were partially assigned based on their exchange cross peaks with their corresponding protons in the major conformer using the ROESY spectrum. The ROESY spectrum was acquired at 0 °C with a spinlock time of 200 ms.

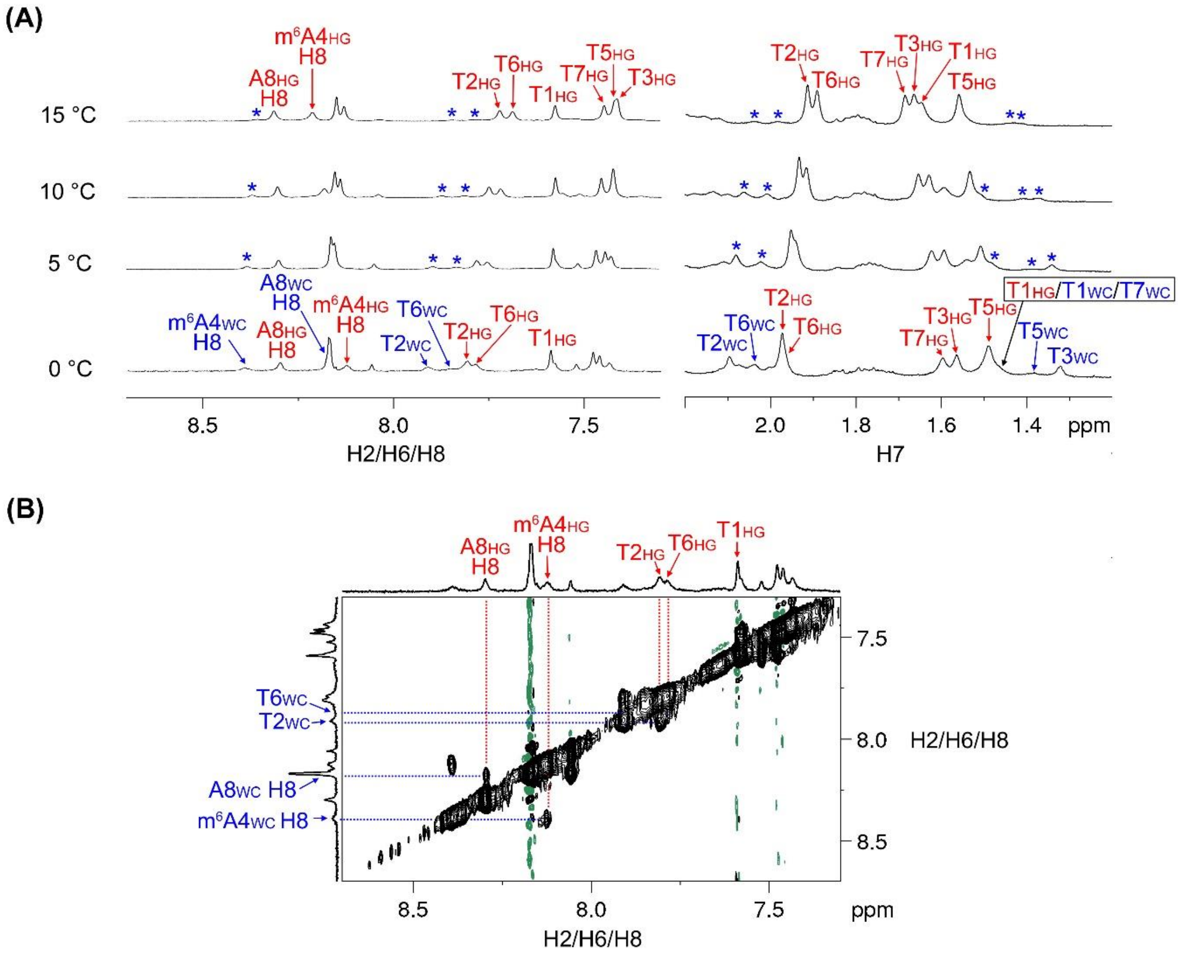

Figure S9. The NMR melting curves constructed by plotting the chemical shifts of T2, T5 and T6 H7 signals as a function of temperature for the major MDB conformer of 5′-TTTm[6]ATTTA-3′. The melting temperature (T_m) was determined to be 10 ± 2 °C by fitting these three melting curves using a two-state transition model as mentioned in Section 4.2 of the main text.

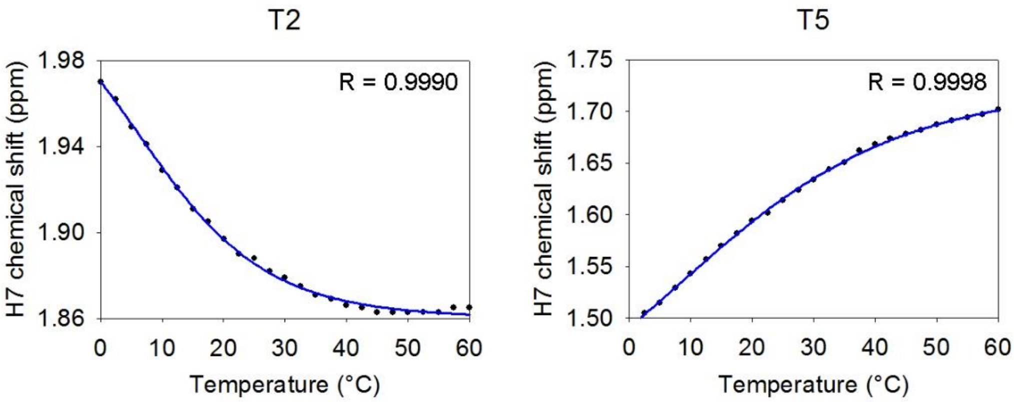

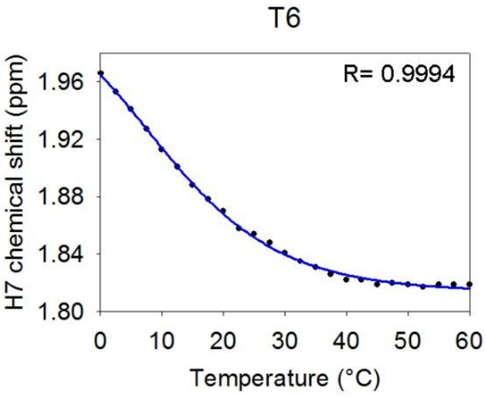

Residue	Fitted T_m (°C)
T2	7.55
T5	12.41
T6	8.99
Average	10 ± 2

Figure S10. The UV heating and cooling profiles of the m¹A MDB overlapped well with each other, suggesting that (i) the unfolding and folding processes appeared to be reversible with the temperature gradient we used, and (ii) m¹A did not convert to m⁶A at high temperatures.

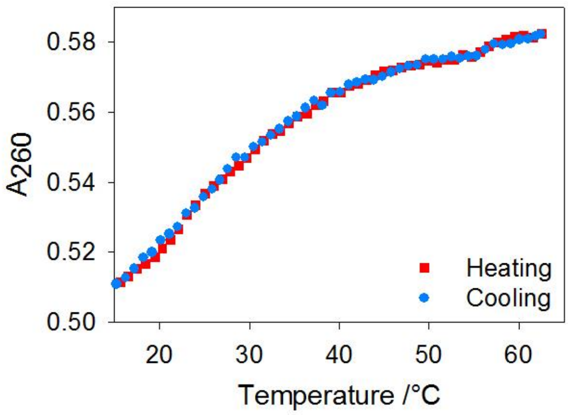

Supplement: Supplementary file 1 [file ijms-22-03633-s001.pdf]
